# Maca (*Lepidium meyenii* Walp*.*) on semen quality parameters: A systematic review and meta-analysis

**DOI:** 10.3389/fphar.2022.934740

**Published:** 2022-08-30

**Authors:** Hye Won Lee, Myeong Soo Lee, Fan Qu, Je-Won Lee, Eunseop Kim

**Affiliations:** ^1^ KM Convergence Research Division, Korea Institute of Oriental Medicine, Daejeon, South Korea; ^2^ KM Science Research Division, Korea Institute of Oriental Medicine, Daejeon, South Korea; ^3^ Women’s Hospital, School of Medicine, Zhejiang University, Hangzhou, Zhejiang, China; ^4^ BM Internal Korean Medicine Clinic, Daegu, South Korea; ^5^ You and Green Korean Medicine Clinic, Daejeon, South Korea

**Keywords:** maca, *Lepidium meyenii* W., semen, infertility, herbal medicine

## Abstract

**Background:** This study aimed to examine the evidence for the effect of *Lepidium meyenii* Walp. [Brassicaceae] (*L. meyenii* W.), known as maca, on improving semen quality.

**Methods:** Nine databases were searched for randomized controlled trials (RCTs) that examined the parameters for improvements in semen quality, regardless of the type of *L. meyenii* W. The risk of bias (ROB) among the studies was evaluated according to the Cochrane ROB tool.

**Results:** Five RCTs met all of the inclusion criteria. Three RCTs showed mixed efficacy of maca in improving semen quality parameters, including sperm concentration and sperm motility, in men experiencing infertility. The meta-analysis also failed to show the efficacy of maca in increasing the sperm concentration compared to the placebo (weighted mean difference, 2.22, 95% confidence interval −2.94 to 7.37, *p* = 0.4). Two other RCTs also showed mixed effects of maca on several semen quality parameters in healthy men.

**Conclusion:** The evidence from the included studies suggests unclear effects of maca on semen quality parameters in both men experiencing infertility and healthy men. However, the total number of RCTs and the total sample size were too small to draw firm conclusions.

## 1 Introduction

Male-specific elements are one of the primary underlying causes of infertility (Sharlip et al., 2002). In this context, semen analysis is a key indicator of challenges that lead to this ailment (Fields et al., 2013). Couples frequently explore complementary and alternative medicine (CAM) to resolve their fertility issues (Bardaweel, 2014; FatemehGhaedi et al., 2020; Choi et al., 2021; Sönmez et al., 2021). According to a recent study, approximately one-fourth of infertile couples seek assistance with at least one form of CAM (Sönmez et al., 2021), particularly herbal products. Another study showed that over 70% of participants used CAM products, among which herbal medicines were the most commonly used (Bardaweel, 2014). One review provides evidence suggesting the efficacy of herbal medicine as a treatment for infertility (Lee et al., 2021).

Between 1,300 and 2,000 years ago, the Andean population appreciated the maca (*Lepidium meyenii* Walp [Brassicaceae]) plant for its nutritional and therapeutic properties and its positive effect on male and female reproductive functions, sexual functions, osteoporosis, depression, anxiety, and energy (Tafuri et al., 2019). One such popular herbal medicine that is used to improve semen quality and treat infertility in general is the maca plant, and most of the experimental data in the literature mainly report the effects of the red, yellow and black hypocotyl types (Gonzales et al., 2004; Gonzales et al., 2006a). Notably, this plant from Peru has long been utilized to enhance sexual functions (Shin et al., 2010; Lee et al., 2011). According to numerous *in vivo* studies, maca is replete with spermatogenic features, which, in turn, positively affect sexual behavior and sperm parameters (Gonzales et al., 2001; Gonzales et al., 2004; Chung et al., 2005; Bogani et al., 2006; Lentz et al., 2006; Rubio et al., 2006; Clément et al., 2010; Clement et al., 2012). One systematic review of the effects of maca on improving semen quality has been published (Lee et al., 2016), and the review includes three randomized controlled trials (RCTs) and 2 uncontrolled clinical trials with several types of controls used for comparisons. This review suggested that maca may be beneficial for improving semen quality. However, this review is now outdated.

Therefore, this article aimed to critically assess the evidence from RCTs on the efficacy of maca to improve semen quality.

## 2 Methods

This protocol is registered at reviewregistry1335 (Lee et al., 2022).

### 2.1 Search of databases

The following databases were searched from inception to April 2022: The Cochrane Central Register of Controlled Trials, EMBASE, PubMed, Virtual Health Library, AMED, KoreaMed, Korean Studies Information, Research Information Service System, and China National Knowledge Infrastructure (CNKI). The strategy related to the search comprised a combination of thesaurus terms and free text. The search terms included “maca OR *Lepidium*” AND “hyposperm OR sperm OR subfertility”. The details of the search strategies for the DBs were given in Supplementary Material. A manual search was conducted of all retrieved articles to ensure their relevance. The search strategy was devoid of any restrictions on language.

### 2.2 Criteria for considering studies

#### 2.2.1 Study design and participants

RCTs that included both infertile and healthy men were included.

#### 2.2.2 Types of interventions and controls

Trials assessing the effects of all maca preparation types, irrespective of their origins, and trials using only maca as the mode of treatment compared to any type of control were included. However, trials that compared varied maca types and any trials with maca as a part of a complex intervention were excluded from the study. Additionally, placebo-controlled trials were included.

#### 2.2.3 Type of outcome measures

Sperm motility and sperm concentration were the primary outcomes. The secondary outcomes were sperm morphology, volume, and counts.

### 2.3 Data extraction and risk-of-bias assessment

The full texts of hard copies of all articles were read. Data were extracted by two independent reviewers according to preset criteria that included methods (comprising the study design, blinding, and follow-up duration), samples (e.g., disease duration, age, size, conditions, and size of population), control treatment, intervention and outcome measures. In addition, the Cochrane risk-of-bias tool was implemented to examine the quality of the included trials (Higgins et al., 2011). Disagreements among reviewers, if any, were resolved through discussion. We assessed the ROB of the included studies, regardless of the publication status. For unpublished reports and abstracts, we used available information or contacted the original authors.

### 2.4 Synthesis of data

Cochrane Collaboration software Review Manager (v.5.4.1) for Windows was used to conduct statistical assessments. An assessment of categorical data was conducted by calculating risk ratios to examine clinical efficacy. Additionally, the mean difference (MD) was calculated to assess continuous data. Continuous and categorical variables were articulated as values of efficacy with 95% confidence intervals (CIs). For instances of varied scales for outcome variables, a decision was made to use the standardized MD over weighted MD (WMD). When heterogeneity (*p* < 0.1 according to the chi-square test and Higgins I^2^ ≥ 50%) was observed, we performed subgroup analyses to ascertain the factor underlying the clinical heterogeneity. Publication bias was assessed using the Egger regression method and by constructing funnel plots. In cases of missing data, incomplete or missing information was sought from the authors of the main study. Albatross plots were also created using STATA/SE v.16.1 (StataCorp LLC, College Station, TX, United States) to visualize the effects of direction on primary and secondary outcomes.

## 3 Results

### 3.1 Study description

**FIGURE 1:**
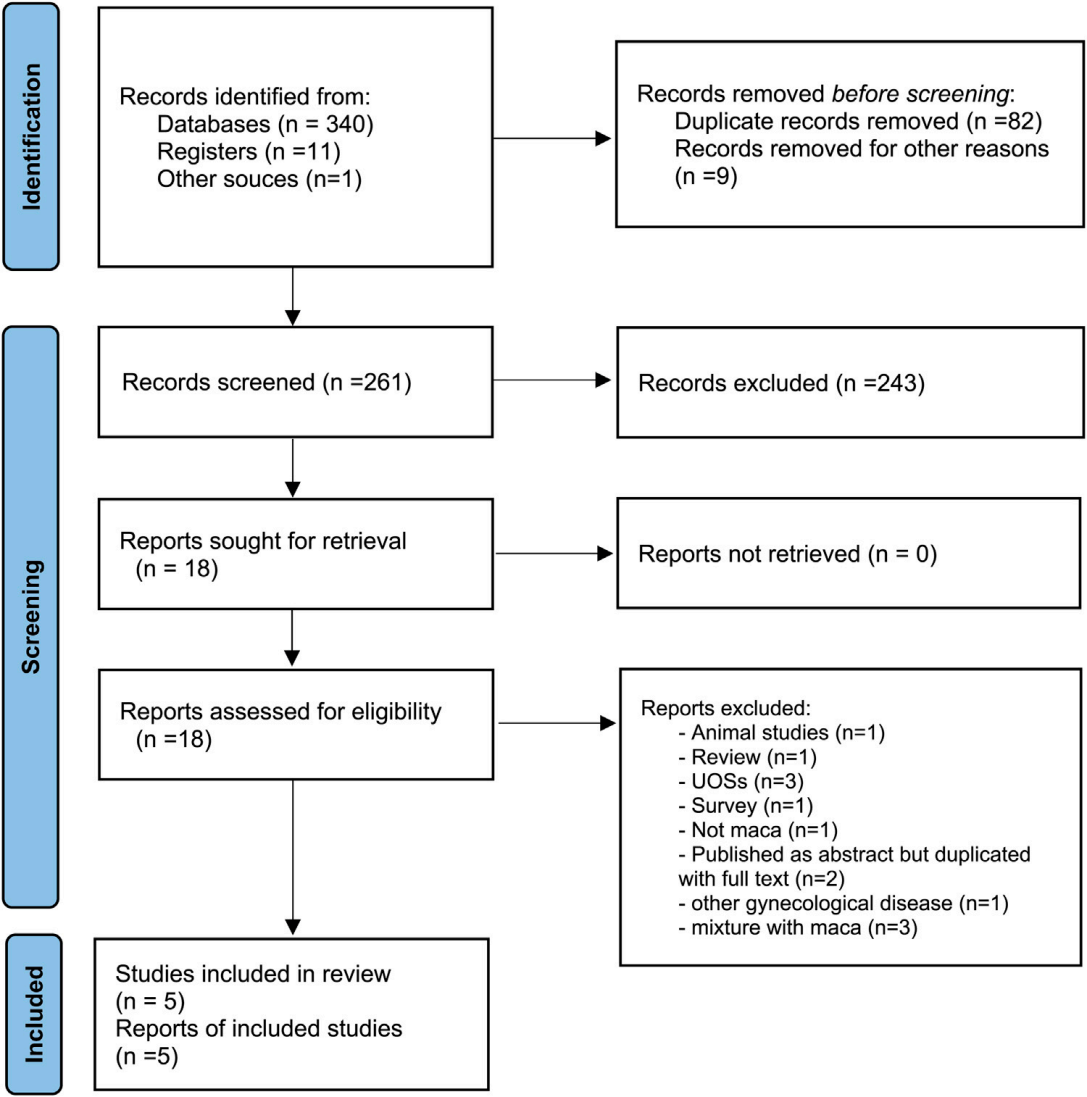
Study flow chart. A flowchart of the patient selection process.

In our search, 352 articles were identified, and five of these met the criteria for inclusion (Kim, 2011; Poveda et al., 2013; Melnikovova et al., 2015; Alcalde and Rabasa, 2020; Melnikovova et al., 2021) (Figure 1; Table 1). Among these, one RCT each was conducted in Spain (Alcalde and Rabasa, 2020), Korea (Kim, 2011), and Panama (Poveda et al., 2013), and two RCTs were conducted in the Czech Republic (Melnikovova et al., 2015; Melnikovova et al., 2021). Across all five studies, maca (1–5 g) was administered orally to the participants. The treatment duration ranged from twelve to 16 weeks. In two studies, the male participants were healthy (Kim, 2011; Melnikovova et al., 2015), while the participants in other studies had problems related to fertility (Poveda et al., 2013; Alcalde and Rabasa, 2020; Melnikovova et al., 2021). Table 2 shows the composition, concentration, use, source, and quality control of maca in the included studies.

**TABLE 1:** Summary of the characteristics of the included studies.

First author (year) location	Design Sample size/condition age (years)	Intervention (regimen)	Control intervention (regimen)	Main outcome measures	Results
Melnikovova (2015) Czech	20 healthy men	(A) *L. meyenii* Walp. (1.75 g/d, for 12 weeks, *n* = 11)	(B) Placebo (milled apple fiber, *n* = 7)	1) Sperm concentration	1) MD, −12.48 [−23.13, −1.83], *p* = 0.02 in favor of B
				2) Total sperm count	2) MD, −22.97 [−102.79, 56.85], NS
				3) Progressively motile sperm count	3) MD, −9.51 [−18.76, −0.26], *p* = 0.04 in favor of B
				4) Motile sperm count	4) MD, −8.55 [−18.35, 1.25], NS
				5) Normal sperm morphology	5) MD, 5.15 [1.13, 7.17], *p* = 0.007 in favor of A
				6) Semen volume	6) MD, 0.55 [0.04, 1.06], *p* = 0.03 in favor of A
Kim (2010) Korea	45 healthy men 30–60	(A) *L. meyenii* Walp. (5 g/d, for 12 weeks, *n* = 15)	(C) Placebo (5 pills/d, n = 15)	Motile sperm count	A + B vs. C, *p* < 0.05 in favor of A + B
		(B) Fermented maca (5 g/d for 12 weeks, *n* = 15)			A vs. C, NS; B vs. C, *p* = 0.03 in favor of B
Poveda (2013) Panama	60 infertile men NR	(A) *L. meyenii* Walp. (1 g/12 h, *n* = 15)	(B) Placebo tablets (1 pill/12 h, n = 15)	1) Sperm concentration	1) NS
			*(C) L-Carnitine (1 pill/12 h, n = 15)*	2) Sperm motility	2) *p* < 0.05 in favor of A
			*(D) Spermotrend (1 pill/8 h, n = 15)*	3) Sperm morphology	3) NS
Melnikovova (2021) Czech	50 infertile men 28–52	(A) *L. meyenii* Walp. (2.8 g/d, for 16 weeks, *n* = 25)	(B) Placebo (milled apple fiber + sucrose, *n* = 25)	1) Sperm concentration	1) MD, -0.38 [-2.35, 1.59], NS
				2) Total sperm count	2) MD, -11.81 [-19.02, -4.60], *p* = 0.001 in favor of B
Alcalde (2020) Spain	69 infertile men 20–40	(A) *L. meyenii* Walp. (2 g/d for 12 weeks, *n* = 33)	(B) Placebo (same color and capsule, *n* = 32)	1) Sperm concentration	1) MD, 4.88 [2.60, 7.16], *p* < 0.0001 in favor of A
				2) Sperm motility	2) MD, -0.33 [-2.07, 1.41], NS
				3) Sperm morphology	3) MD, 0.85 [-0.17, 1.87], NS
				4) Semen volume	4) MD, 0.05 [-0.20, 0.30], NS

MD: mean difference; NA: not available; NR: not reported; NS: not significant. The italicized parts were not considered in the analysis. Maca; *L. meyenii* Walp. [Brassicaceae].

**TABLE 2:** Compositions, concentration, usage, source, and quality control of maca.

First author (year)	Name of preparation	Concentration	Source	Quality control	Chemical analysis	Components
Melnikovova (2015)	Gelatinized yellow maca (*L. meyenii* Walp.)	1.75 g/d	Peruvian company Andean Roots, Ltd. (harvested in the Peruvian Andes)	Hospital Preparation	HPLC-DAD analytical system	Macamides (methoxy-n-benzyl-(9Z.12Z.15Z)-octadecatrienamide, n-benzyl-(9Z.12Z.15Z)-octadecatrienamide, methoxy-n-benzyl-(9Z.12Z)-octadecadienamide, n-benzyl-(9Z.12Z)-octadecadienamide, n-benzylhexadecanamide, and n-benzyl-(9Z)-octadecanamide)
Kim (2010)	Gelatinized yellow maca (*L. meyenii* Walp.)	5 g/d	Cabex S.A.	Prepared according to the Ministry of Food and Drug Safety	HPLC-DAD analytical system	n-benzyl-hexadecanamide
Poveda (2013)	Maca (*L. meyenii* Walp.) extracts	1 g/12 h	Nature’s Way Product, Inc	NR	NR	NR
Melnikovova (2021)	Gelatinized yellow maca (*L. meyenii* Walp.)	2.8 g/d	Peruvian company Andean Roots, Ltd	Hospital Preparation	HPLC-DAD analytical system	Macamides (unknown macamide, linolenic acid, n-benzyl-(9Z, 12Z, 15Z)-octadecatrienamide, linoleic acid, n-benzyl-(9Z, 12Z)-octadecadienamide, n-benzylpentadecanamide, n-(3-methoxybenzyl)-hexadecanamide, n-benzylhexadecanamide, n-benzyl-(9Z)-octadecenamide, n-benzylheptadecanamide, n-benzyloctadecanamide, methoxy-derivatives)
Alcalde (2020)	Gelatinized maca (*L. meyenii* Walp.)	2 g/d	NR	NR	NR	NR

HPLC-DAD, high-performance liquid chromatography with a diode-array detection; NR, not reported.

### 3.2 Risk of bias

**FIGURE 2:**
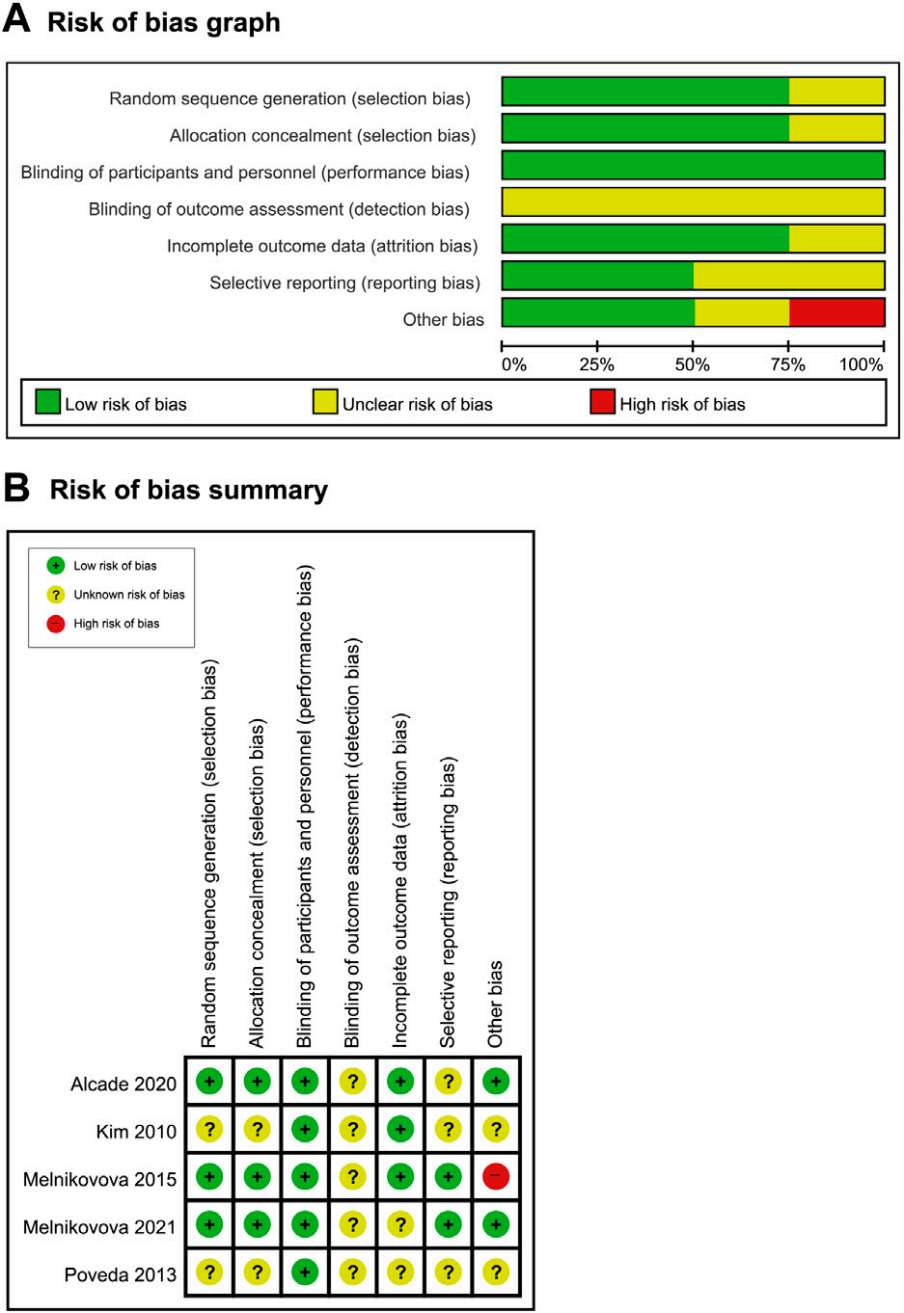
**(A)** Risk-of-bias graph and **(B)** risk-of-bias summary: the present authors’ judgments about the risk of bias for each item in all included studies.

Three RCTs reported random sequence generation methods and employed allocation concealment (Melnikovova et al., 2015; Alcalde and Rabasa, 2020; Melnikovova et al., 2021) (Figure 2). The double-blind design was used in all five studies, but the baseline between the three groups was significantly different (Melnikovova et al., 2015; Alcalde and Rabasa, 2020; Melnikovova et al., 2021). Notably, one RCT was published in the form of an abstract, and most of the domains were unclear (Poveda et al., 2013). Additionally, the other RCT was a report that remained unpublished, and the majority of domains were unclear (Kim, 2011).

### 3.3 Effects of interventions

#### 3.3.1 Men with infertility

**FIGURE 3:**
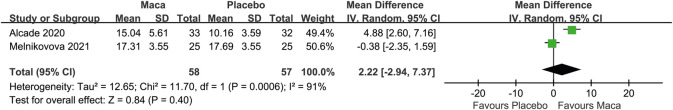
Forest plot of the effect of maca on the semen concentration.

Among the three aforementioned studies (Poveda et al., 2013; Alcalde and Rabasa, 2020; Melnikovova et al., 2021), one RCT indicated that maca exerted a positive effect on the sperm concentration, but the other two studies did not report a similar outcome (Poveda et al., 2013; Melnikovova et al., 2021). The meta-analysis also failed to show the efficacy of maca in increasing the sperm concentration compared to the placebo (WMD, 2.22, 95% CI -2.94 to 7.37, *p* = 0.4, I^2^ = 91%, Figure 3). The effects of maca on sperm motility were evaluated in two RCTs. One study showed favorable effects of maca, but the other one did not. Regarding sperm morphology, no major difference was observed between the placebo and the herb.

#### 3.3.2 Healthy men

The effect of maca on semen quality in healthy men was assessed in two RCTs and compared to a placebo (Kim, 2011; Melnikovova et al., 2015). One RCT did not report any positive effects of the herb on either sperm concentration or motile/total sperm count (Melnikovova et al., 2015). The other study suggested a mixed effect of maca on sperm motility (Kim, 2011).

#### 3.3.3 Adverse events

One RCT listed the reason for withdrawal due to adverse events (Melnikovova et al., 2021). However, no trial tried to examine the adverse effects of maca.

### 3.4 Albatross plot

**FIGURE 4:**
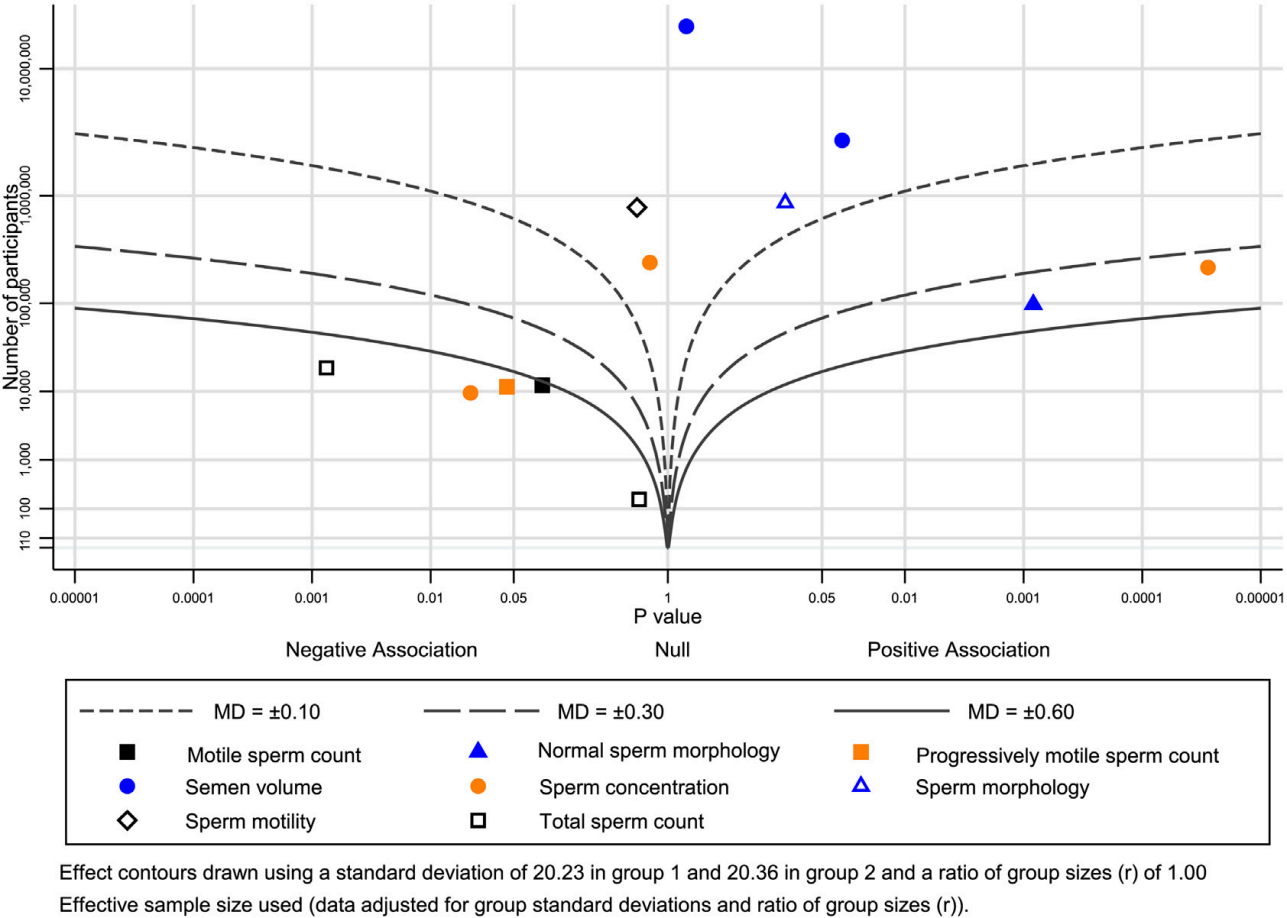
Albatross plot of the main outcomes.

The albatross plot showing the effects of direction and size range by *p* value and a particular sample size was generated for each included study (Figure 4, different outcome groups are shown in different colors). The points for the subjective data were scattered across the contour lines (Figure 4). All points were on the side of a positive association, indicating that maca exerts a positive effect on semen quality parameters.

## 4 Discussion

### 4.1 Summary of findings

Few trials examining the effects of maca on semen quality parameters have been conducted. The majority of trials that have been conducted have yet to reveal any positive effect of the herb on the aforementioned issue. Three of the five trials suggested that the efficacy of maca in improving semen quality parameters in infertile men was mixed (Poveda et al., 2013; Alcalde and Rabasa, 2020; Melnikovova et al., 2021). The same findings were evident even among healthy men in two other RCTs (Kim, 2011; Melnikovova et al., 2015)[19,20]. Overall, the evidence indicates the ambiguous effect of maca on semen quality parameters among both healthy and infertile men.

One of the five RCTs assessed in the systematic review was published as an abstract (Poveda et al., 2013), while another one was an unpublished report (Kim, 2011), and both studies had not undergone peer review. Both studies were also lacking in details of reporting. We contacted the authors and interviewed them but were unable to retrieve the full reports. One RCT was related to a commercial company, and hidden conflicts of interest regarding their results may be present (Kim, 2011). In three RCTs, significant differences in the baseline values of some semen parameters were reported (Melnikovova et al., 2015; Alcalde and Rabasa, 2020; Melnikovova et al., 2021).

### 4.2 Differences from previous reviews

The aim of our review was to further increase the existing body of evidence by adding new RCTs of maca for improving semen quality. Unlike prior studies, this study was able to identify two novel RCTs with low bias-related risks (Alcalde and Rabasa, 2020; Melnikovova et al., 2021), thus updating the evidence. The results of our review are slightly different from one previous review showing that maca may be beneficial for improving semen quality.

### 4.3 Experimental evidence from *in vivo* and *in vitro* studies

Possible mechanisms of action might assume significance based on the assumption that maca helped improve semen quality parameters. Maca products, such as raw materials and their extracts, have frequently been investigated in sperm production models and have produced beneficial effects in preclinical studies. According to multiple studies involving rodents and horses, among other models, maca intake affected the reproductive health of males, especially in terms of spermatogenesis disorders and quantitative sperm parameters.

Quantitative sperm parameters refer to the analysis of the sperm volume, concentration, total count, motility, and strict morphology, regardless of whether abnormalities are present in the sperm. Several experimental studies have investigated the potential effects of maca on increasing sperm production. Semen samples collected from stallions during breeding that were treated with yellow maca food supplementation indicated a positive effect on the following parameters: ejaculate volumes, sperm concentration, total and progressive motility, acrosome integrity, elongation of the spermatozoa head, and the percentage of spermatozoa with fragmented DNA (Tafuri et al., 2019; D'Anza et al., 2021; Del Prete et al., 2018). Several studies of rodent models found that maca and its extracts improved the acrosome reaction, sperm motility, and count through the increased structural and functional preservation of Leydig cells, which produce testosterone (Valdivia Cuya et al., 2016; Onaolapo et al., 2018; Aoki et al., 2019). The potential benefits of maca are related to its androgen-like effects on counteracting CYP-induced changes in the male reproductive system (Onaolapo et al., 2018).

Spermatogenesis describes the development of haploid spermatozoa from germ cells in the seminiferous tubules of the testes (Ray et al., 2014). According to one study, the oral administration of maca to rodent models increased spermiation stages (VII-VIII), germ cell mitosis (IX-XI), the epididymal sperm count, and daily sperm production (Gonzales et al., 2013). In particular, black maca is proposed to be more helpful in terms of increasing epididymal sperm motility (stages II-VI and VIII) and total sperm count, while the duration of stage VIII was reported to increase after the administration of red and yellow maca (Gonzales et al., 2006a; Gonzales et al., 2006b). Maca increased the length of stages VII-VIII in the seminiferous tubules; on the other hand, the cauda epididymal sperm count, sperm motility, and serum estradiol level were not affected by any of the doses studied (Chung et al., 2005). The progression of spermatogenesis in those who received maca had a longer duration of stages IX-XI for sperm epithelium, and the duration of stages XII-XIV, which are related to sperm epithelium, were also increased (Gonzales et al., 2003).

### 4.4 The gap between clinical and experimental results

The results of animal studies revealed positive effects of maca on semen quality, but our review obtained limited evidence for the effectiveness of maca in infertility. This difference is because humans and rodent models are fundamentally different in body structure and metabolism. Although many factors are controlled in animal studies, they are conducted without fully considering important aspects of disease development, such as diet, lifestyle, environmental factors, stress, and psychological/social factors. Even if the genes of an animal are similar to that of a human, its physiological functions may differ due to differences in base sequence, amino acid and protein structure, and responses to drug metabolism may also be different in humans.

### 4.5 Limitations

This study is not free from some limitations. For example, although our extensive searches included Korean/Chinese/English databases, the selection of all relevant articles cannot be guaranteed. In particular, manufacturers of maca supported several studies, which may have resulted in inherent bias. Most industry-sponsored studies yielded positive results. Each of the studies included in this review obtained the powder from a company associated with a particular maca product. In addition, standardization of herbal medicines, including maca, is a limited process because the composition of the phytocomplex depends on several factors, such as cultivation methods, environmental and weather conditions, place of cultivation, ecotype considered, preparation methods and preservation of the herbal supplement. In addition, the included studies used different treatment doses and courses. These differences would lead to large heterogeneity, and the results should be interpreted with caution. The lack of primary data and their less than optimal quality were other limitations of this study.

One might wonder why the data from only two studies should be pooled. The main reasons for performing meta-analyses are to increase power, improve precision, answer questions that were not answered by individual studies, resolve controversies due to conflicting results, and develop new hypotheses (Deeks et al., 2019). A meta-analysis can be performed by combining 2 or more studies. However, the use of statistics does not guarantee that the results are valid. Therefore, in our case, the conclusions must remain tentative.

One argument for including a publication presented as only an abstract and an unpublished report is that obtaining and including data from unpublished studies seems to be an obvious method to avoid publication bias, but including data from unpublished studies may itself lead to bias (Sterne et al., 2008). Another issue is the willingness of researchers of unpublished studies to provide data (Sterne et al., 2008). We tried to obtain complete information by contacting, interviewing or calling the investigators (Kim, 2011). However, we were unable to obtain this information. In the case of the unpublished study, we called the investigators and KFDA to obtain the full report but did not receive the results. We also met with the CEO of the company manufacturing this product, but he was unwilling to provide us with full reports. This lack of information may reduce the strength of the conclusions of this study.

### 4.6 Implications for future research

In the future, more stringent methods and standards of testing must be incorporated to better understand how maca affects the parameters of semen quality. Factors such as sample size, standardization of product, and optimal dosage of treatment, among others, must be considered thoroughly. CONSORT guidelines must be followed during the process of conducting clinical trials in future studies.

## 5 Conclusion

The current systematic review did not provide overwhelming evidence for the efficacy of maca in improving the parameters of semen quality in both infertile and healthy men. Nevertheless, a better informed conclusion can be drawn only after increasing the sample size, improving the methodological quality of the primary studies, and conducting more rigorous RCTs in the future.

## Data Availability

The original contributions presented in the study are included in the article/Supplementary Material, further inquiries can be directed to the corresponding author.

## Abbreviation

CAM, complementary and alternative medicine; CIs, confidence intervals; CONSORT, CONsolidated Standards Of Reporting Trials; *L. meyenii*, *Lepidium meyenii*; RCT, randomized controlled trial; ROB, risk of bias; WMD, weighted mean difference.
