# Supplementary material for: Maca (Lepidium meyenii Walp.) on semen quality parameters: A systematic review and meta-analysis
Source: Front Pharmacol. 2022 Aug 30;13:934740. doi: 10.3389/fphar.2022.934740 (PMC9468664; doi:10.3389/fphar.2022.934740)
Supplement: Supplementary file 1 [file DataSheet1.PDF]

Supplement 1. Database search strategy

1. PubMed Search Strategy

#1 “Lepidium” [MeSH Terms]

#2 “Lepidium meyenii” [tiab]

#3 “maca” [tiab]

#4 #1-#3/OR

#5 “Infertility” [MeSH Terms]

#6 “Semen” [MeSH Terms]

#7 “Sperm Count” [MeSH Terms]

#8 “Sperm Motility” [MeSH Terms]

#9 “Subfertility” [Text Word]

#10 “Sperm” [Text Word]

#11 “Sperm Volume” [Text Word]

#12 “Sperm Density” [Text Word]

#13 “Sperm Concentration” [Text Word]

#14 “Sperm Morphology” [Text Word]

#15 #5-#14/OR

#16 #4 AND #15

2. Cochran library Search Strategy

#1. “Lepidium” ti,ab,kw
#2. “Lepidium meyenii” ti,ab,kw
#3. “maca” ti,ab,kw
#4. MeSH descriptor [Lepidium] explode all trees

#5. #1-#4/OR

#6. “sperm”ti, ab, kw

#7. “semen”ti, ab, kw

#8. MeSH descriptor [infertility] explode all trees

#9. #6- #8/OR

#10. #5 AND #9

3. EMBASE Search Strategy

S1: ti(“Lepidium”) OR ab(“Lepidium”)

S2: ti(“Lepidium meyenii”) OR ab(“Lepidium meyenii”)

S3: ti(“maca”) OR ab(“maca”)

S4: ti(“sperm”) OR ab(“sperm”)

S5: ti(“semen”) OR ab(“semen”)

S6: ti(“infertility”) OR ab(“infertility”)

S7: ti(“subfertility”) OR ab(“subfertility”)

S8: S3 OR S2 OR S1

S9: S7 OR S6 OR S5 OR S4

S10. S9 AND S10
